# Constructivist Learning Theory–Based Teaching Methods in Nursing Education in China: Protocol for a Systematic Review and Meta-Analysis

**DOI:** 10.2196/93097

**Published:** 2026-07-16

**Authors:** Jun Yang, Jiejie Ge, Mengyuan Li, Chuanying Zhang, Sijing Peng

**Affiliations:** 1 School of Nursing, Anhui University of Chinese Medicine, Xinzhan District, Longzihu Road No. 350, Hefei, Anhui, 230012, China, 86 13675687458; 2 Key Laboratory of Geriatric Nursing and Health, School of Nursing, Anhui University of Chinese Medicine, Hefei, Anhui, China

**Keywords:** constructivist learning theory, nursing education, teaching methods, China, systematic review, meta-analysis

## Abstract

**Background:**

The cognitive paradigm in medical education is undergoing a transition from traditional knowledge transmission to learner-centered knowledge construction. In China, this shift is aligned with the *Outline of the Plan for the Construction of China into an Education Powerhouse* (*2024‐2035*), which mandates high-quality, intrinsic development in nursing curricula. While constructivist learning theory (CLT)–based teaching methods (eg, problem-based learning, case-based learning, and situational simulation) have been widely explored across Chinese nursing institutions, the evidentiary base remains geographically fragmented and methodologically heterogeneous. A systematic synthesis is required to inform national, evidence-based educational reforms.

**Objective:**

This protocol describes a systematic review and meta-analysis designed to evaluate the effectiveness of CLT-based teaching methods vs traditional lecture-based models on Chinese nursing students’ theoretical knowledge, practical skills, self-directed learning ability, and critical thinking disposition.

**Methods:**

A comprehensive systematic search will be conducted across 9 electronic databases: PubMed, Web of Science, the Cochrane Library, Embase, CINAHL, China National Knowledge Infrastructure, Wanfang Data, VIP Database (Chinese Scientific and Technological Journal Database), and China Biology Medicine. The search period spans from database inception to September 27, 2025, with a planned update through June 11, 2026, before final synthesis. Randomized controlled trials and quasi-experimental studies involving Chinese nursing students will be included. Two independent reviewers will screen records, perform full-text assessment, extract data using standardized forms, and code composite CLT interventions, digital or technology-enhanced components, and cluster- or class-based designs using prespecified decision rules. Risk of bias will be assessed using the Cochrane Risk of Bias tool 2 (RoB 2) for randomized trials and the Joanna Briggs Institute critical appraisal tools for quasi-experimental studies. Meta-analysis will be performed using RevMan 5.4 and Stata 18.0, with random-effects models and prespecified subgroup and sensitivity analyses.

**Results:**

This protocol was finalized in February 2026. A preliminary systematic search conducted on September 27, 2025, identified 990 records before deduplication. As of February 6, 2026, deduplication had been completed and title and abstract screening had been initiated. Data extraction, risk-of-bias assessment, and statistical synthesis had not yet started at the protocol stage and will be conducted only after completion of the updated search, final study selection, and full-text eligibility assessment. The final search update was scheduled through June 11, 2026, before data synthesis. The results manuscript will be submitted after completion of all prespecified review steps, with the timeline depending on the number and complexity of newly identified studies.

**Conclusions:**

This review will provide a robust evidentiary foundation for the strategic deployment of constructivist methodologies in Chinese nursing education, specifically addressing the needs of vocational and undergraduate programs in the era of digital transformation.

## Introduction

The cognitive paradigm in medical education has undergone a profound transformation, shifting from a linear transmission of knowledge to a dynamic process of individual meaning-making. This evolution is epitomized by constructivist learning theory (CLT), which posits that learners actively build their knowledge systems through interaction with specific sociocultural contexts, collaboration with peers, and scaffolded guidance from instructors [1-3]. In the domain of nursing education, where clinical reasoning and humanistic care are paramount, CLT-based pedagogical models—such as problem-based learning (PBL), case-based learning (CBL), and anchored instruction—have emerged as vital alternatives to traditional lecture-based models [4].

In China, this pedagogical shift is now intrinsically linked to the national strategic mandate. The recently promulgated *Outline of the Plan for the Construction of China into an Education Powerhouse* (*2024‐2035*) emphasizes a transition from “scale expansion” to “high-quality, intrinsic development” [5]. Within this policy framework, nursing education is increasingly tasked with fostering “complex clinical decision-making skills” rather than rote memorization. Recent evidence suggests that CLT-based “project-driven teaching” in foundational nursing courses significantly enhances students’ humanistic care abilities and self-directed learning competencies compared with conventional methods [6,7]. Furthermore, as the Chinese health care sector embraces “industry-education integration,” there is a critical need to construct “school-hospital-enterprise” ecosystems where students construct knowledge within authentic clinical scenarios [6,8].

Despite the proliferation of localized studies on CLT in Chinese nursing programs, the evidence base remains geographically fragmented and methodologically heterogeneous. Current innovations often focus on specific curricula—such as the application of the BOPPPS (bridge-in, objectives, preassessment, participatory learning, postassessment, and summary) model in nursing ethics [9]—yet lack a rigorous, systematic synthesis of their collective impact across different educational tiers (eg, vocational vs undergraduate).

Furthermore, the rapid digitalization of the 2025‐2026 educational landscape, characterized by the uptake of virtual simulation and other technology-enhanced learning modalities, has expanded the range of constructivist teaching strategies implemented in nursing programs [10,11]. This systematic review and meta-analysis protocol is designed to address these gaps. By synthesizing high-quality evidence from Chinese nursing education, this study aims to provide a robust evidentiary foundation for evidence-based curriculum reform and the strategic deployment of constructivist methodologies in the digital age. However, this expansion has also blurred the boundary between constructivist pedagogy and broader active learning approaches. Composite educational interventions, digitally mediated learning activities, and class-based implementation designs are common in nursing education studies but are not always reported with sufficient methodological detail. A prespecified protocol is therefore needed to ensure transparent eligibility judgments, reproducible classification of interventions, and appropriate synthesis decisions.

## Methods

### Study Design and Registration

This systematic review and meta-analysis protocol was prepared in accordance with the PRISMA-P (Preferred Reporting Items for Systematic Review and Meta-Analysis Protocols) guidelines [12,13] and is registered with PROSPERO (CRD420251159499). Any substantive amendments to the protocol, including changes to search dates, intervention classification, or analysis methods, will be documented in PROSPERO when applicable and reported in the final review.

### Eligibility Criteria

We will use the PICO (population, intervention, comparator, outcome) framework to define the following inclusion and exclusion criteria.

#### Population

The review will include nursing students currently enrolled in accredited educational programs in China (including mainland China, Hong Kong, Macao, and Taiwan). No restrictions will be placed on the level of education (secondary vocational, higher vocational, undergraduate, or postgraduate).

#### Interventions

Eligible interventions will include teaching methodologies that are explicitly grounded in CLT or that clearly operationalize core constructivist principles. For the purposes of this review, an intervention will be judged as CLT-based when the study description demonstrates at least one of the following features: learner-centered knowledge construction, collaborative problem-solving, contextualized or authentic learning tasks, scaffolding by instructors, reflective learning, inquiry-based learning, or learning activities embedded in simulated or real clinical scenarios. Interventions will not be classified as CLT-based solely because they use active learning terminology; the pedagogical rationale or implementation process must indicate a constructivist orientation. Eligible CLT-based strategies may include PBL, CBL, scenario-based simulation, anchored instruction, scaffolding teaching, project-based learning, and other explicitly constructivist educational approaches.

#### Classification of Composite CLT Interventions

Composite interventions will be eligible if the constructivist component is explicitly described and constitutes a substantive part of the pedagogical design. For classification, 2 reviewers will code each intervention according to the dominant constructivist strategy reported by the original authors and reflected in the intervention procedures. If a study combines 2 or more CLT strategies with comparable emphasis (eg, PBL plus CBL or scenario simulation plus project-based learning), it will be coded as a composite CLT intervention rather than being forced into a single category. Such studies will be included in the overall synthesis. For subgroup analyses, composite interventions will be analyzed separately when sufficient studies are available; otherwise, their influence will be examined through sensitivity analyses.

#### Definition of Digital or Technology-Enhanced CLT

Digital or technology-enhanced CLT will be defined as an intervention in which digital tools are integral to the delivery of constructivist learning activities. Examples include online collaborative discussion, learning management system–based inquiry tasks, app-supported group learning, virtual simulation, virtual reality scenarios, or other digital platforms that support problem-solving, interaction, reflection, or scenario-based learning. Routine use of Microsoft PowerPoint slides, classroom multimedia displays, or administrative online communication will not by itself be classified as digital or technology-enhanced CLT. When the proportion of digital delivery is reported, interventions in which at least half of the structured learning activities are delivered or completed through digital platforms will be coded as predominantly digital; otherwise, classification will be based on the role of digital tools in the intervention design and adjudicated by 2 reviewers.

#### Comparators

The control group must receive traditional teaching methods, primarily defined as teacher-centered, lecture-based learning, or conventional demonstration-based instruction.

#### Outcomes

Studies must report at least one of the following primary or secondary outcomes:

- The primary outcomes will include theoretical knowledge scores (standardized examination results) and practical clinical skill scores (objective structured clinical examination or checklist-based assessments).
- The secondary outcomes will include self-directed learning ability, critical thinking disposition, professional identity, and humanistic care competency.

#### Study Design

We will include randomized controlled trials (RCTs) and quasi-experimental studies (eg, non-RCTs). Qualitative studies, case reports, editorials, and reviews will be excluded.

### Search Strategy

A comprehensive systematic search will be conducted across 9 electronic databases: PubMed, Web of Science, the Cochrane Library, Embase, CINAHL, China National Knowledge Infrastructure (CNKI), Wanfang Data, VIP, and China Biology Medicine (CBM). The search period spans from database inception to September 27, 2025, with a planned update through June 11, 2026, before final synthesis. In addition, we will screen the reference lists of included studies and relevant reviews to identify additional eligible records.

Search terms will include a combination of controlled vocabulary terms and free-text keywords, including constructivism- and strategy-related terms (eg, “constructivism,” “constructivist learning theory,” “problem-based learning,” “case-based learning,” “project-based learning,” “scaffolding,” “anchored instruction,” “scenario-based simulation,” “situational simulation,” “virtual simulation,” and “virtual reality”) combined with population and setting terms (eg, “nursing education,” “nursing students,” “China,” and “Chinese”). Database-specific field strategies will be used to improve retrieval sensitivity. For English-language databases, controlled vocabulary terms and free-text terms will be combined where available. For Chinese databases, search fields will be adapted to the structure of each database. In CNKI and Wanfang Data, searches will be conducted using topic fields where available, covering title, abstract, and keywords. In VIP and CBM, equivalent title, abstract, and keyword or subject fields will be used. The full search strategies for all databases, including the PubMed strategy and database-specific adaptations, are provided in Multimedia Appendix 1.

### Selection and Data Extraction

Two researchers (JY and ML) will independently screen titles and abstracts against the predefined criteria using EndNote (Clarivate Analytics). Full-text versions of potentially eligible studies will then be retrieved for final eligibility assessment. Disagreements will be resolved through consensus or consultation with a third reviewer (SP). The study selection procedure will be documented according to the PRISMA (Preferred Reporting Items for Systematic Reviews and Meta-Analyses) 2020 flow diagram after completion of the updated search and full-text screening [14].

Data will be extracted using a standardized, pilot-tested form. Extracted information will include the following:

- Study metadata: first author, publication year, and geographical location
- Participants: sample size, educational level, and baseline characteristics
- Intervention details: type of CLT-based method, constructivist features, dominant or composite CLT strategy, digital or technology-enhanced components, duration, intensity, and setting (classroom, online, simulation, or clinical)
- Design and analysis features: unit of allocation, class- or cluster-based design, adjustment for clustering, reported intracluster correlation coefficient (ICC), timing of outcome measurement, and handling of repeated measures
- Outcomes: mean scores, SDs, and specific measurement tools used

### Quality Assessment

Two reviewers will independently assess the risk of bias. For RCTs, the Cochrane Risk of Bias (RoB 2) tool [15] will be used, focusing on 5 domains: randomization process, deviations from intended interventions, missing outcome data, measurement of the outcome, and selection of the reported result. For cluster-randomized trials, the cluster-specific considerations in RoB 2 will be applied where relevant. For quasi-experimental studies, we will use the Joanna Briggs Institute critical appraisal tools [16].

### Statistical Analysis

#### Overview

Meta-analysis will be performed using RevMan (version 5.4; Cochrane) and Stata (version 18.0; StataCorp LLC). For continuous variables, results will be summarized as standardized mean differences (SMDs) with 95% CIs. When studies report the same outcome using the same scale, mean differences will be considered where appropriate; otherwise, SMDs will be used to improve comparability across measurement tools [17]. RCTs and quasi-experimental studies will first be analyzed separately by study design when sufficient studies are available for a given outcome. If the interventions, populations, outcomes, and measurement time points are judged to be sufficiently comparable, an overall random-effects meta-analysis including both RCTs and quasi-experimental studies will also be conducted. The influence of quasi-experimental studies will be examined through sensitivity analyses restricted to RCTs, and differences between study designs will be explored through design-based subgroup analyses when data permit.

#### Assessment of Heterogeneity

Statistical heterogeneity will be quantified using the *I*^2^ statistic and the Cochran *Q* test. An *I*^2^ value of >50% will be considered to represent substantial heterogeneity. Given the inherent diversity in educational interventions, a random-effects model will be the primary analytical approach [17,18].

#### Unit-of-Analysis Issues

Because educational interventions are often allocated at the class, course, or teaching-group level, we will prespecify procedures for cluster- or class-based designs. When included studies use cluster randomization or class-based allocation, we will preferentially extract effect estimates that account for clustering. If clustering has not been accounted for in the original analysis, we will adjust the effective sample size using a design effect when the ICC and average cluster size are available [17,19,20]. If the ICC is not reported, we will contact the corresponding authors. If no ICC information can be obtained, we will conduct sensitivity analyses using plausible ICC values derived from comparable educational studies and report the assumptions explicitly. Studies with unadjusted cluster designs will also be considered in sensitivity analyses to assess the influence of potential unit-of-analysis errors. For studies reporting repeated measurements, the immediate postintervention outcome will be used as the primary end point; follow-up outcomes will be analyzed separately when sufficient data are available.

### Subgroup and Sensitivity Analyses

To explore potential sources of heterogeneity, subgroup analyses will be conducted based on (1) educational level (undergraduate vs vocational), (2) dominant intervention type (eg, PBL, CBL, scenario-based simulation, project-based learning, or composite CLT intervention), (3) delivery mode (digital or technology-enhanced CLT vs predominantly face-to-face CLT), and (4) geographical region within China (eastern vs central or western regions) to reflect regional educational disparities. Composite interventions will be analyzed as a separate subgroup when sufficient studies are available; otherwise, their influence will be examined in sensitivity analyses.

Sensitivity analyses will be performed by sequentially omitting individual studies to evaluate the robustness of the pooled estimates. Additional sensitivity analyses will include analyses restricted to RCTs to examine the influence of quasi-experimental studies, exclusion of studies with unadjusted cluster or class-based allocation, exclusion of composite CLT interventions when appropriate, and exclusion of studies using imputed or assumed ICC values. If sufficient studies are available for a given outcome (typically ≥10), we will consider meta-regression to explore whether effect estimates vary by prespecified study-level characteristics, including educational level, intervention duration, intervention type, delivery mode, study design, and unit of allocation.

### Publication Bias and Certainty of Evidence

If the meta-analysis includes ≥10 studies for a specific outcome, publication bias will be assessed using funnel plots and the Egger regression test (*P*<.05 indicating significant bias). The certainty of the evidence for each outcome will be evaluated using the Grading of Recommendations Assessment, Development and Evaluation (GRADE) approach [21].

### Ethical Considerations

This study is a systematic review and meta-analysis of published literature and does not involve collecting individual-level personal data or assigning interventions; therefore, ethics approval and informed consent are not required. Findings will be disseminated through peer-reviewed publication and conference presentations.

## Results

The development of this protocol was finalized in February 2026. A preliminary systematic search was conducted on September 27, 2025, which identified a total of 990 records across the targeted electronic databases before deduplication. As of February 6, 2026, the research team has completed the deduplication process and initiated the first stage of title and abstract screening.

The final search update was scheduled through June 11, 2026, before data synthesis. Data extraction, risk-of-bias assessment, and statistical synthesis had not yet started at the protocol stage and will be conducted only after completion of the updated search, final study selection, and full-text eligibility assessment. Because the updated search may identify additional eligible studies, the subsequent stages will be completed sequentially rather than according to a fixed short interval. The final results manuscript will be submitted after these steps are completed, with the anticipated timeline extending to late 2026 or early 2027, depending on the number and complexity of newly identified studies.

**Figure 1.**
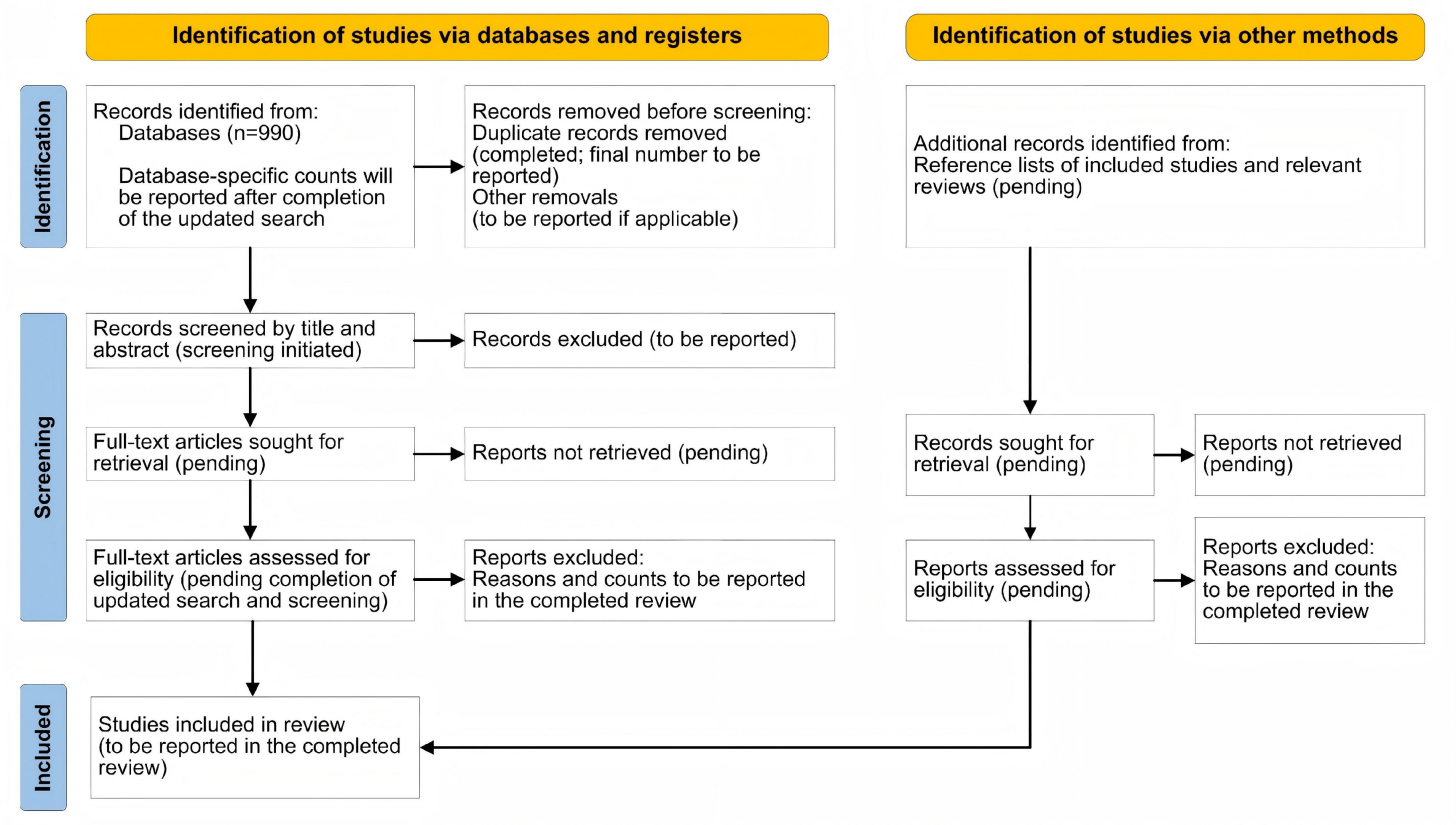
Protocol-stage PRISMA (Preferred Reporting Items for Systematic Reviews and Meta-Analyses) 2020 flow diagram for the planned study selection process.

Figure 1 presents the protocol-stage PRISMA 2020 flow diagram for the planned study selection process. At this stage, the preliminary search identified 990 records before deduplication, deduplication has been completed, and title and abstract screening has been initiated. The final numbers of records screened, excluded, assessed for eligibility, and included in the review will be reported in the completed systematic review after the updated search, full-text assessment, and final study selection are completed.

## Discussion

### Principal Considerations

To our knowledge, this systematic review and meta-analysis will provide the first meta-analytic synthesis of CLT-based teaching methodologies within the specific context of Chinese nursing education. Although individual studies have examined student-centered learning approaches, the absence of an evidence synthesis has limited their translation into broader curriculum planning and implementation.

Our anticipated findings may clarify how constructivist elements, such as meaning construction and collaborative dialogue, relate to the development of higher-order competencies in nursing students. In recent Chinese nursing education reforms, competencies such as clinical reasoning, self-directed learning, and humanistic care have received increasing attention as educational priorities [9,11,22]. By examining the effects of PBL, CBL, project-driven models, simulation-based strategies, and composite CLT interventions, this review may assist educators in selecting pedagogical approaches that are appropriate for different educational tiers.

### Comparison With Prior Work

Previous reviews suggest that active learning approaches, simulation-supported learning, and virtual reality–based educational strategies can improve selected learning outcomes in nursing education [4,23-28]. However, many syntheses do not explicitly account for sociocultural and institutional features of Chinese nursing programs, where teacher-centered hierarchies may remain influential. In addition, our review is situated within the 2024‐2025 industry-education integration reform agenda that promotes school-hospital-enterprise collaboration to bridge the theory-practice gap [6,8]. This focus will allow the review to reflect recent policy directions toward competency-based education and vocational training reform in China [4,5,8,29,30].

### Strengths and Limitations

A major strength of this protocol is its adherence to the PRISMA-P statement and related guidance [12,13] and the inclusion of quasi-experimental studies, which are frequently used in educational settings where pure randomization is logistically challenging. In response to the methodological complexity of educational intervention research, this protocol prespecifies rules for judging CLT-based interventions, coding composite interventions, classifying digital or technology-enhanced delivery, and handling cluster- or class-based designs. These procedures are expected to improve the transparency and reproducibility of the planned review.

However, certain limitations must be acknowledged. First, restricting inclusion to Chinese- and English-language studies may introduce language bias. Second, heterogeneity in intervention components (eg, duration, intensity, instructor expertise, constructivist features, delivery mode, and outcome measurement) may limit the interpretability of pooled effects. Third, some educational intervention studies may use class-based allocation without adequately adjusting for clustering, which could introduce unit-of-analysis concerns. To address these issues, we will apply random-effects models, prespecified subgroup analyses, cluster-related adjustment procedures where possible, and sensitivity analyses; where sufficient studies are available, exploratory meta-regression may be conducted to examine potential sources of variation.

### Conclusions and Policy Implications

This review will provide an evidentiary foundation to support the ongoing high-quality development of nursing education in China. By synthesizing the effects of CLT-based teaching strategies on knowledge acquisition, practical skills, and learner-centered outcomes (eg, self-directed learning and critical thinking), the findings may inform curriculum planning and implementation at vocational and undergraduate levels. The results are expected to support the broader shift from rote memorization toward constructivist approaches that prepare nursing students for contemporary clinical practice [17,30].

## Supplementary material

10.2196/93097 Multimedia Appendix 1 Full search strategies for all databases.

10.2196/93097 Checklist 1 PRISMA-P checklist.

## Abbreviations

BOPPPS: bridge-in, objectives, preassessment, participatory learning, postassessment, and summary
CBL: case-based learning
CBM: China Biology Medicine
CLT: constructivist learning theory
CNKI: China National Knowledge Infrastructure
GRADE: Grading of Recommendations Assessment, Development and Evaluation
ICC: intracluster correlation coefficient
PBL: problem-based learning
PICO: population, intervention, comparator, outcome
PRISMA: Preferred Reporting Items for Systematic Reviews and Meta-Analyses
PRISMA-P: Preferred Reporting Items for Systematic Review and Meta-Analysis Protocols
RCT: randomized controlled trial
RoB 2: Risk of Bias 2
SMD: standardized mean difference
