# Supplementary material for: Constructivist Learning Theory–Based Teaching Methods in Nursing Education in China: Protocol for a Systematic Review and Meta-Analysis
Source: JMIR Res Protoc. 2026 Jul 16;15:e93097. doi: 10.2196/93097 (PMC13375208; doi:10.2196/93097)
Supplement: Multimedia Appendix 1 [file resprot-v15-e93097-s001.pdf]

Multimedia Appendix 1. Full Search Strategies for All Databases

Constructivist Learning Theory–Based Teaching Methods in Chinese Nursing Education: Protocol for a Systematic Review and Meta-analysis

This appendix provides database-specific search strategies for the planned systematic review and meta-analysis. Search results are not prefilled and should be completed after actual database testing. The search period is from database inception to June 11, 2026. No study-design filter is applied at the search stage; study design will be assessed during screening according to the eligibility criteria.

Search Concept Framework

Table 0. Search concept framework.

Concept group	English terms	Chinese terms	Search principle
**Concept 1**: Constructivist learning theory and related teaching methods	constructivism; constructivist learning theory; constructivist teaching; constructivist pedagogy; problem-based learning; PBL; case-based learning; CBL; project-based learning; project-driven; scaffolding; anchored instruction; inquiry-based learning; scenario-based simulation; situational simulation; simulation-based learning; virtual simulation; virtual reality; VR; flipped classroom; blended learning	建构主义；建构主义学习理论；建构主义教学；建构主义教学模式；问题导向学习；PBL；案例教学；案例导向学习；CBL；项目教学；项目驱动；支架式教学；抛锚式教学；探究式学习；情景模拟；情境模拟；模拟教学；虚拟仿真；虚拟现实；翻转课堂；混合式教学	A high-recall strategy is used. Final inclusion as a CLT-based intervention will be judged during screening according to prespecified constructivist features.
**Concept 2**: Nursing education and nursing students	nursing education; nursing student*; student nurse*; students, nursing; undergraduate nurs*; vocational nursing; nursing curriculum; nursing teaching; clinical nursing education	护理教育；护理教学；护理课程；护理学生；护生；护理本科生；高职护理；中职护理；临床护理教学；基础护理教学	This concept group is retained in all databases.
**Concept 3**: China-related context	China; Chinese; mainland China; Hong Kong; Macao; Macau; Taiwan	中国；我国；国内；内地；中国香港；中国澳门；中国台湾	This concept group is retained in English-language databases. It is not routinely added to Chinese databases to avoid over-restricting retrieval.

Full Search Strategies

Table S1. Search strategy for PubMed/MEDLINE.

#Step	Search Terms and Boolean Logic	Results
#1	("Constructivism"[tiab] OR constructivis*[tiab] OR "constructivist learning theor*"[tiab] OR "constructivist teaching"[tiab] OR "constructivist pedagogy"[tiab] OR "problem-based learning"[tiab] OR PBL[tiab] OR "case-based learning"[tiab] OR CBL[tiab] OR "project-based learning"[tiab] OR "project-driven"[tiab] OR scaffolding[tiab] OR "anchored instruction"[tiab] OR "inquiry-based learning"[tiab] OR "scenario-based simulation"[tiab] OR "situational simulation"[tiab] OR "simulation-based learning"[tiab] OR "virtual simulation"[tiab] OR "virtual reality"[tiab] OR VR[tiab] OR "flipped classroom"[tiab] OR "blended learning"[tiab] OR "Problem-Based Learning"[Mesh] OR "Simulation Training"[Mesh] OR "Patient Simulation"[Mesh] OR "Virtual Reality"[Mesh])	101,932
#2	("Education, Nursing"[Mesh] OR "Students, Nursing"[Mesh] OR "nursing education"[tiab] OR "nursing student*"[tiab] OR "student nurse*"[tiab] OR "undergraduate nurs*"[tiab] OR "vocational nursing"[tiab] OR "nursing curriculum"[tiab] OR "nursing teaching"[tiab] OR "clinical nursing education"[tiab])	121,888
#3	("China"[Mesh] OR China[tiab] OR Chinese[tiab] OR "mainland China"[tiab] OR "Hong Kong"[tiab] OR Macao[tiab] OR Macau[tiab] OR Taiwan[tiab])	826,701
#4	#1 AND #2 AND #3	256

Filters: English and Chinese; publication date from database inception to June 11, 2026; no study-design filter was applied at the search stage.

Table S2. Search strategy for Web of Science Core Collection.

#Step	Search Terms and Boolean Logic	Results
#1	TS=("constructivism" OR constructivis* OR "constructivist learning theor*" OR "constructivist teaching" OR "constructivist pedagogy" OR "problem-based learning" OR PBL OR "case-based learning" OR CBL OR "project-based learning" OR "project-driven" OR scaffolding OR "anchored instruction" OR "inquiry-based learning" OR "scenario-based simulation" OR "situational simulation" OR "simulation-based learning" OR "virtual simulation" OR "virtual reality" OR VR OR "flipped classroom" OR "blended learning")	220,186
#2	TS=("nursing education" OR "nursing student*" OR "student nurse*" OR "students nursing" OR "undergraduate nurs*" OR "vocational nursing" OR "nursing curriculum" OR "nursing teaching" OR "clinical nursing education")	41,863
#3	TS=(China OR Chinese OR "mainland China" OR "Hong Kong" OR Macao OR Macau OR Taiwan)	1,905,822
#4	#1 AND #2 AND #3	198

Filters: English and Chinese; timespan from database inception to June 11, 2026; document type not restricted at the search stage; no study-design filter was applied.

Table S3. Search strategy for Cochrane Library.

#Step	Search Terms and Boolean Logic	Results
#1	[mh "Problem-Based Learning"] OR [mh "Simulation Training"] OR [mh "Patient Simulation"] OR [mh "Virtual Reality"] OR (constructivism OR constructivis* OR (constructivist NEXT learning NEXT theor*) OR (constructivist NEXT teaching) OR (constructivist NEXT pedagogy) OR (problem NEXT based NEXT learning) OR PBL OR (case NEXT based NEXT learning) OR CBL OR (project NEXT based NEXT learning) OR project-driven OR scaffolding OR (anchored NEXT instruction) OR (inquiry NEXT based NEXT learning) OR (scenario NEXT based NEXT simulation) OR (situational NEXT simulation) OR (simulation NEXT based NEXT learning) OR (virtual NEXT simulation) OR (virtual NEXT reality) OR VR OR (flipped NEXT classroom) OR (blended NEXT learning)):ti,ab,kw	16,031
#2	[mh "Education, Nursing"] OR [mh "Students, Nursing"] OR ((nursing NEXT education) OR (nursing NEXT student*) OR (student NEXT nurse*) OR (undergraduate NEXT nurs*) OR (vocational NEXT nursing) OR (nursing NEXT curriculum) OR (nursing NEXT teaching) OR (clinical NEXT nursing NEXT education)):ti,ab,kw	4,850
#3	[mh "China"] OR (China OR Chinese OR (mainland NEXT China) OR (Hong NEXT Kong) OR Macao OR Macau OR Taiwan):ti,ab,kw	75,825
#4	#1 AND #2 AND #3	64

Filters: English and Chinese; date from database inception to June 11, 2026; no study-design filter was applied at the search stage.

Table S4. Search strategy for Embase

#Step	Search Terms and Boolean Logic	Results
#1	('problem based learning'/exp OR 'simulation training'/exp OR 'patient simulation'/exp OR 'virtual reality'/exp OR constructivism:ti,ab,kw OR constructivis*:ti,ab,kw OR 'constructivist learning theor*':ti,ab,kw OR 'constructivist teaching':ti,ab,kw OR 'constructivist pedagogy':ti,ab,kw OR 'problem-based learning':ti,ab,kw OR pbl:ti,ab,kw OR 'case-based learning':ti,ab,kw OR cbl:ti,ab,kw OR 'project-based learning':ti,ab,kw OR 'project-driven':ti,ab,kw OR scaffolding:ti,ab,kw OR 'anchored instruction':ti,ab,kw OR 'inquiry-based learning':ti,ab,kw OR 'scenario-based simulation':ti,ab,kw OR 'situational simulation':ti,ab,kw OR 'simulation-based learning':ti,ab,kw OR 'virtual simulation':ti,ab,kw OR 'virtual reality':ti,ab,kw OR vr:ti,ab,kw OR 'flipped classroom':ti,ab,kw OR 'blended learning':ti,ab,kw)	140,064
#2	('nursing education'/exp OR 'nursing student'/exp OR 'nursing education':ti,ab,kw OR 'nursing student*':ti,ab,kw OR 'student nurse*':ti,ab,kw OR 'undergraduate nurs*':ti,ab,kw OR 'vocational nursing':ti,ab,kw OR 'nursing curriculum':ti,ab,kw OR 'nursing teaching':ti,ab,kw OR 'clinical nursing education':ti,ab,kw)	130,603
#3	('china'/exp OR china:ti,ab,kw OR chinese:ti,ab,kw OR 'mainland China':ti,ab,kw OR 'Hong Kong':ti,ab,kw OR macao:ti,ab,kw OR macau:ti,ab,kw OR taiwan:ti,ab,kw)	7,031,906
#4	#1 AND #2 AND #3	244

Filters: English and Chinese; date from database inception to June 11, 2026; no study-design filter was applied at the search stage.

Table S5. Search strategy for CINAHL/EBSCO.

#Step	Search Terms and Boolean Logic	Results
S1	(MH "Problem-Based Learning+" OR MH "Simulation+" OR MH "Virtual Reality+" OR TI ("constructivism" OR constructivis* OR "constructivist learning theor*" OR "constructivist teaching" OR "constructivist pedagogy" OR "problem-based learning" OR PBL OR "case-based learning" OR CBL OR "project-based learning" OR "project-driven" OR scaffolding OR "anchored instruction" OR "inquiry-based learning" OR "scenario-based simulation" OR "situational simulation" OR "simulation-based learning" OR "virtual simulation" OR "virtual reality" OR VR OR "flipped classroom" OR "blended learning") OR AB ("constructivism" OR constructivis* OR "constructivist learning theor*" OR "constructivist teaching" OR "constructivist pedagogy" OR "problem-based learning" OR PBL OR "case-based learning" OR CBL OR "project-based learning" OR "project-driven" OR scaffolding OR "anchored instruction" OR "inquiry-based learning" OR "scenario-based simulation" OR "situational simulation" OR "simulation-based learning" OR "virtual simulation" OR "virtual reality" OR VR OR "flipped classroom" OR "blended learning"))	128,548
S2	(MH "Education, Nursing+" OR MH "Students, Nursing+" OR TI ("nursing education" OR "nursing student*" OR "student nurse*" OR "undergraduate nurs*" OR "vocational nursing" OR "nursing curriculum" OR "nursing teaching" OR "clinical nursing education") OR AB ("nursing education" OR "nursing student*" OR "student nurse*" OR "undergraduate nurs*" OR "vocational nursing" OR "nursing curriculum" OR "nursing teaching" OR "clinical nursing education"))	163,661
S3	(MH "China+" OR TI (China OR Chinese OR "mainland China" OR "Hong Kong" OR Macao OR Macau OR Taiwan) OR AB (China OR Chinese OR "mainland China" OR "Hong Kong" OR Macao OR Macau OR Taiwan))	1,653,356
S4	S1 AND S2 AND S3	226

Filters: English and Chinese; date from database inception to June 11, 2026; no study-design filter was applied at the search stage.

Table S6. Search strategy for CNKI (China National Knowledge Infrastructure).

#Step	Search Expression	Results
#1	TKA=('建构主义' + '建构主义学习理论' + '建构主义教学' + '建构主义教学模式' + '问题导向学习' + 'PBL' + '案例教学' + '案例导向学习' + 'CBL' + '项目教学' + '项目驱动' + '支架式教学' + '抛锚式教学' + '探究式学习' + '情景模拟' + '情境模拟' + '模拟教学' + '虚拟仿真' + '虚拟现实' + '翻转课堂' + '混合式教学')	441,822
#2	TKA=('护理教育' + '护理教学' + '护理课程' + '护理学生' + '护生' + '护理本科生' + '护理专业学生' + '高职护理' + '中职护理' + '临床护理教学' + '基础护理教学')	77,992
#3	#1 AND #2	8212

Filters: Publication date from database inception to June 11, 2026; language: Chinese and English; no study-design filter was applied at the search stage.

Table S7. Search strategy for Wanfang Data.

#Step	Search Expression	Results
#1	主题:("建构主义" OR "建构主义学习理论" OR "建构主义教学" OR "建构主义教学模式" OR "问题导向学习" OR "PBL" OR "案例教学" OR "案例导向学习" OR "CBL" OR "项目教学" OR "项目驱动" OR "支架式教学" OR "抛锚式教学" OR "探究式学习" OR "情景模拟" OR "情境模拟" OR "模拟教学" OR "虚拟仿真" OR "虚拟现实" OR "翻转课堂" OR "混合式教学")	605,225
#2	主题:("护理教育" OR "护理教学" OR "护理课程" OR "护理学生" OR "护生" OR "护理本科生" OR "高职护理" OR "中职护理" OR "临床护理教学" OR "基础护理教学")	187,193
#3	主题:("建构主义" OR "建构主义学习理论" OR "建构主义教学" OR "建构主义教学模式" OR "问题导向学习" OR "PBL" OR "案例教学" OR "案例导向学习" OR "CBL" OR "项目教学" OR "项目驱动" OR "支架式教学" OR "抛锚式教学" OR "探究式学习" OR "情景模拟" OR "情境模拟" OR "模拟教学" OR "虚拟仿真" OR "虚拟现实" OR "翻转课堂" OR "混合式教学") and 主题:("护理教育" OR "护理教学" OR "护理课程" OR "护理学生" OR "护生" OR "护理本科生" OR "高职护理" OR "中职护理" OR "临床护理教学" OR "基础护理教学")	11,142

Filters: Publication date from database inception to June 11, 2026; language: Chinese and English; no study-design filter was applied at the search stage.

Table S8. Search strategy for VIP Database.

#Step	Search Expression	Results
#1	M=("建构主义" OR "建构主义学习理论" OR "建构主义教学" OR "建构主义教学模式" OR "问题导向学习" OR "PBL" OR "案例教学" OR "案例导向学习" OR "CBL" OR "项目教学" OR "项目驱动" OR "支架式教学" OR "抛锚式教学" OR "探究式学习" OR "情景模拟" OR "情境模拟" OR "模拟教学" OR "虚拟仿真" OR "虚拟现实" OR "翻转课堂" OR "混合式教学")	307,622
#2	M=("护理教育" OR "护理教学" OR "护理课程" OR "护理学生" OR "护生" OR "护理本科生" OR "高职护理" OR "中职护理" OR "临床护理教学" OR "基础护理教学")	67,493
#3	M=("建构主义" OR "建构主义学习理论" OR "建构主义教学" OR "建构主义教学模式" OR "问题导向学习" OR "PBL" OR "案例教学" OR "案例导向学习" OR "CBL" OR "项目教学" OR "项目驱动" OR "支架式教学" OR "抛锚式教学" OR "探究式学习" OR "情景模拟" OR "情境模拟" OR "模拟教学" OR "虚拟仿真" OR "虚拟现实" OR "翻转课堂" OR "混合式教学") AND M=("护理教育" OR "护理教学" OR "护理课程" OR "护理学生" OR "护生" OR "护理本科生" OR "高职护理" OR "中职护理" OR "临床护理教学" OR "基础护理教学")	7,104

Filters: Publication date from database inception to June 11, 2026; language: Chinese and English; no study-design filter was applied at the search stage.

Table S9. Search strategy for SinoMed/CBM (recommended strategy).

#Step	Search Expression	Results
#1	("建构主义"[常用字段] OR "建构主义学习理论"[常用字段] OR "建构主义教学"[常用字段] OR "建构主义教学模式"[常用字段] OR "问题导向学习"[常用字段] OR "PBL"[常用字段] OR "案例教学"[常用字段] OR "案例导向学习"[常用字段] OR "CBL"[常用字段] OR "项目教学"[常用字段] OR "项目驱动"[常用字段] OR "支架式教学"[常用字段] OR "抛锚式教学"[常用字段] OR "探究式学习"[常用字段] OR "情景模拟"[常用字段] OR "情境模拟"[常用字段] OR "模拟教学"[常用字段] OR "虚拟仿真"[常用字段] OR "虚拟现实"[常用字段] OR "翻转课堂"[常用字段] OR "混合式教学"[常用字段])	71,164
#2	("护理教育"[常用字段] OR "护理教学"[常用字段] OR "护理课程"[常用字段] OR "护理学生"[常用字段] OR "护生"[常用字段] OR "护理本科生"[常用字段] OR "高职护理"[常用字段] OR "中职护理"[常用字段] OR "临床护理教学"[常用字段] OR "基础护理教学"[常用字段])	65,990
#3	#1 AND #2	6,207

Filters: Publication date from database inception to June 11, 2026; language: Chinese and English; no study-design filter was applied at the search stage.
